# 

**Published:** 1903-01

**Authors:** 


### CONTENTS.

ORIGINAL ARTICLES:
- Vitality and Immunity. By Carl Schulin, M.D. . . . 1
- A Historical Essay on the Relation of Veterinary Medicine to the Medical Profession. By S. G. Burkholder, M.D., M.D.V. . . . 7
- Arecoline Hydrobromate. By Walter Lincoln Bell, D.V.S. . . . 12
- Hemorrhagic Septicæmia. By M. H. Reynolds, M.D., V.M. . . . 15

ABSTRACTS FROM FOREIGN JOURNALS . . . 30

REPORTS OF CASES:
- Myxoma of the Nasal Fossa of a Horse. By John J. Repp, V.S. . . . 35

EDITORIALS:

- Our New Staff . . . 36

- The Compensation of Veterinarians for Directing Public Work . . . 36

NECROLOGY . . . 38

COMMUNICATIONS . . . 39

SANITARY CONTROL WORK . . . 40
- Measures to Eradicate Foot-and-Mouth Disease . . . 43

BOOK REVIEWS . . . 45

COMMENCEMENT EXERCISES . . . 46

SOCIETY PROCEEDINGS—NOTES—PERSONALS . . . 47–63

